# Lung ultrasound detects regional aeration inhomogeneity in ventilated preterm lambs

**DOI:** 10.1038/s41390-023-02781-1

**Published:** 2023-08-17

**Authors:** Laura L. H. He, Gillian Foo, Kelly R. Kenna, Ellen Douglas, Monique Fatmous, Rebecca J. Sutton, Elizabeth J. Perkins, Magdy Sourial, Prue M. Pereira-Fantini, David G. Tingay, Arun Sett

**Affiliations:** 1 Neonatal Research, Murdoch Children’s Research Institute, Victoria, VIC Australia; 2 Department of Paediatrics, University of Melbourne, Victoria, VIC Australia; 3 Joan Kirner Women’s and Children’s Hospital, Western Health, Victoria, VIC Australia; 4 Translational Research Unit, Murdoch Children’s Research Institute, Victoria, VIC Australia; 5 Newborn Research Centre, The Royal Women’s Hospital, Victoria, VIC Australia; 6 Department of Obstetrics and Gynaecology, The University of Melbourne, Victoria, VIC Australia

## Abstract

**Background:**

Inhomogeneous lung aeration is a significant contributor to preterm lung injury. EIT detects inhomogeneous aeration in the research setting. Whether LUS detects inhomogeneous aeration is unknown. The aim was to determine whether LUS detects regional inhomogeneity identified by EIT in preterm lambs.

**Methods:**

LUS and EIT were simultaneously performed on mechanically ventilated preterm lambs. LUS images from non-dependent and dependent regions were acquired and reported using a validated scoring system and computer-assisted quantitative LUS greyscale analysis (Q-LUS_MGV_). Regional inhomogeneity was calculated by observed over predicted aeration ratio from the EIT reconstructive model. LUS scores and Q-LUS_MGV_ were compared with EIT aeration ratios using one-way ANOVA.

**Results:**

LUS was performed in 32 lambs (~125d gestation, 128 images). LUS scores were greater in upper anterior (non-dependent) compared to lower lateral (dependent) regions of the left (3.4 vs 2.9, *p* = 0.1) and right (3.4 vs 2.7, *p* < 0.0087). The left and right upper regions also had greater LUS scores compared to right lower (3.4 vs 2.7, *p* < 0.0087) and left lower (3.7 vs 2.9, *p* = 0.1). Q-LUS_MGV_ yielded similar results. All LUS findings corresponded with EIT regional differences.

**Conclusion:**

LUS may have potential in measuring regional aeration, which should be further explored in human studies.

**Impact:**

- Inhomogeneous lung aeration is an important contributor to preterm lung injury, however, tools detecting inhomogeneous aeration at the bedside are limited.
- Currently, the only tool clinically available to detect this is electrical impedance tomography (EIT), however, its use is largely limited to research.
- Lung ultrasound (LUS) may play a role in monitoring lung aeration in preterm infants, however, whether it detects inhomogeneous lung aeration is unknown.
- Visual LUS scores and mean greyscale image analysis using computer assisted quantitative LUS (Q-LUS_MGV_) detects regional lung aeration differences when compared to EIT.
- This suggests LUS reliably detects aeration inhomogeneity warranting further investigation in human trials.

## Introduction

Neonatal respiratory distress syndrome (RDS) secondary to surfactant deficiency is the most frequent acute respiratory disorder faced by infants born preterm.^1^ Complex secondary insults including mechanical trauma and resultant lung injury may lead to bronchopulmonary dysplasia (BPD), a leading cause of neonatal mortality and morbidity.^1^ Although RDS is classically described as a homogeneous disease, this simply refers to the type of lung disease. Within the lung, the resultant volume states including aeration are inhomogeneous. Ineffective aeration in the first few hours after birth can further enhance regional differences in lung aeration, which may contribute to lung injury.^2–4^ Therefore, early detection of inhomogeneous lung aeration may help better guide respiratory support at birth and potentially reduce subsequent lung injury.

Tools to measure regional lung aeration in infants are limited. Electrical impedance tomography (EIT) and lung ultrasound (LUS) are radiation-free lung imaging tools which have established a strong role in neonatal research. EIT is a non-invasive bedside lung imaging modality that reliably detects lung volume changes and has been validated against computed tomography (CT).^4–6^ EIT delivers small electrical currents through the chest via circumferential electrodes and measures the received currents in the corresponding electrodes. As electrical impedance increases with lung aeration, the measured impedance can be used to calculate relative changes in lung aeration.^6^ Although EIT accurately provides regional aeration and ventilation information of the lung, it is currently predominantly limited to clinical research in infants.^6–8^

LUS is a quick, inexpensive lung imaging tool.^9–11^ The interaction of ultrasound with the pleural surface generates reproducible artefact patterns that vary proportionally with lung aeration.^12^ In neonatal research, these artefact patterns are assigned numerical values and combined to form a semi-quantitative LUS score.^13^ Whilst LUS has been shown to reliably diagnose RDS and predict BPD, its ability to detect inhomogeneous lung aeration has not been explored.^3,13,14^

Despite widespread use, visually graded, categorical scoring systems are operator dependent and lack the resolution to detect small changes in lung volume.^15,16^ To overcome this, quantitative methods to analyse LUS images are being explored.^17^ The greyness of ultrasound images is measured using computer assisted image analysis (Quantitative lung ultrasound mean grey value (Q-LUS_MGV_), providing objective quantification of LUS image characteristics. We have recently demonstrated that Q-LUS_MGV_ can detect small changes in lung volume in preterm lambs.^17^ However, whether this measure can detect inhomogeneous lung aeration is unknown.

We hypothesised that LUS would detect regional aeration differences in preterm lambs. To explore this, we compared regional differences in aeration as determined by differences in the classical visual LUS scoring system and Q-LUS_MGV,_ to measurements of lung aeration derived from EIT in preterm lambs managed with standardised mechanical ventilation.

## Methods

This study was part of a larger group of studies investigating impacts of different respiratory support strategies on the initiation of lung injury after preterm birth. This study was approved by the Murdoch Children’s Research Institute Animal Ethics Committee, Melbourne, Australia (approval number: A940) and reported as per the Animal Research: Reporting of In-Vivo Experiments (ARRIVE) guidelines.^18^

### Animal preparation

Betamethasone treated, Border-Leicester/Merino ewes were anaesthetised, and preterm lambs (124–128 days gestations [term: 145 days]) were partially exteriorised for instrumentation with carotid artery and jugular vein catheters, and intubation with a 4.0 mm cuffed endotracheal tube. Following this, a custom-built 32-electrode EIT belt (Swisscom AG, Landquart, Switzerland) was fitted around the chest.^19,20^ The lamb was then fully exteriorised from the uterus and lung liquid was passively drained via the endotracheal tube immediately prior to mechanical ventilation (SLE5000, SLE Ltd, Croydon, UK) as per the primary study protocol. Immediately on commencing mechanical ventilation a 180-s dynamic positive end expiratory pressure (PEEP) manoeuvre to transient maximum PEEP of 14 cm H_2_O as previously described was performed during pressure-controlled, time-cycled, volume-targeted ventilation in the supine position.^19^ Thereafter mechanical ventilation continued with PEEP 8 cm H_2_O, maximal peak inspiratory pressure (P_max_) 50 cmH_2_O, respiratory rate 60 breaths per minute. Tidal volume (V_t_) was set at 7 mL/kg. Ventilation was ceased at 15 min and the endotracheal tube clamped. After a further 30 min of apnoeic placental support to allow for development of molecular markers of lung injury,^21^ the static pressure volume relationship of the respiratory system was mapped using the super-syringe method as previously described,^19^ and a lethal dose of sodium pentobarbitone (100 mg/kg) was administered. Sedation and anaesthesia to a level that suppressed breathing was maintained throughout via the mother. A heat lamp, warm towels and heating mats were used to prevent hypothermia.

### Electrical impedance tomography (EIT)

EIT images were sampled at 48 frames/second. Data was reconstructed using an anatomically correct finite element model of the lamb thorax filtered to the respiratory domain.^20^ Volumes of the dorsal, central, and ventral regions were determined from weighting pixel distributions of each region to calibrate whole lung volumes. The aeration state of each region was determined by calculating the ratio of the measured relative aeration in a region to the anatomical size of that region compared to whole lung based on the finite element model (that is the number of pixels included in that lung region relative to the total pixels for the whole lung).^6^ Lung aeration is a continuous measure but they are categorised by the following thresholds for clinically relevant interpretation: <1.0; relative underinflated, 1.0; homogenous aeration, >1.0; relative hyperinflation. Aeration from the ventral and central regions of left and right lung were calculated and compared.^19,22^

### Lung ultrasound (LUS)

LUS was performed at 15 min while lambs were mechanically ventilated using a Logiq E (GE Healthcare, Wauwatosa, WI; System 1, *n* = 21) and Terason USMART 3200 T (Terason, Burlington, MA; System 2, *n* = 11) ultrasound system with a 12-megahertz linear transducer. Depth was set at 2.5 cm and the focal zone positioned at the pleural line. Gain was set to 60 decibels and not adjusted between animals or ultrasound systems. Images were obtained from the right and left upper anterior and lower lateral regions in supine position. Based on the shape on the lamb’s chest, this corresponds to the ventral and central EIT regions. Randomized, de-identified LUS images were scored by an investigator G.F. (2 years LUS experience) who was not present during the experiment and blinded to the ventilation settings and lung volume measurements. LUS images were scored using our LUS scoring system that we have previously validated against gold standard measures of lung volume in this animal model.^15^ In brief, the scoring system is based on categorical artefact patterns and ranges from 0–5, where 0 indicates complete loss of aeration (a fully hepatized appearance) and 5 indicates the best aeration representing a normal well aerated lung (normal A-line profile). As we have previously reported excellent inter-observer variability with our scoring system,^15^ this was not repeated.

### Computer assisted grey scale analysis

Computer assisted grey scale analysis was performed using our previously described methods.^17^ As images were acquired using two ultrasound systems, animals from each group were analysed separately to account for differences between systems, where uncompressed LUS images were de-identified, imported into FIJI, Image J (National Institute of Health, Bethesda, Maryland) in DICOM format and converted to 8-bit format for greyscale analysis. The region of interest (ROI) was delineated as previously reported.^17^ Q-LUS_MGV_ ranges from 0 (black) to 255 (white) and was measured in arbitrary units (A.U.).

### Statistical analysis

As this was a sub-study of a larger group of studies, a convenience sample of 32 consecutive lambs were consecutively studied if the operator (A.S.) was available. Assuming a standard deviation in aeration in LUS scores of 1.0, this sample size provided 97% power to detect difference in LUS scores of 1.0 between the ventral and central regions (G*Power Version 3.1.9.6, Mannheim, Germany).^23^ The left central and ventral regions of EIT were compared to the left lower lateral and upper anterior regions of LUS, respectively. The same was compared on the right side of the lung (right central vs right lower lateral; right ventral vs right upper anterior), as seen in Supplementary Fig. 1. Significant differences within EIT and LUS measures were calculated by one-way ANOVA with repeated measures and Tukey’s post hoc correction for multiple comparisons. Statistical significance was set at <0.05. Analysis was performed using GraphPad Prism (V9.4.1, GraphPad Software, San Diego).

## Results

128 LUS recordings were obtained from 32 lambs. Table 1 details the lamb characteristics. No lambs had evidence of foetal distress or acidosis. No studied lambs were excluded from the final analysis.

**Table 1:** Lamb characteristics (*n* = 32).

Gestational age, days	125 (1)		
Male *n* (%)	22 (69%)		
Birth weight, g	3000 (470)		
Lung fluid drained (ml/kg)	35 (12)		
Blood gas analysis	Cord	5 min	15 min
pH	7.36 (0.06)	7.40 (0.07)	7.40 (0.07)
pCO_2_ (mm Hg)	43.4 (4.0)	35.9 (4.1)	36.1 (4.5)
Base excess (mmol/l)	−1 (3.5)	−2.6 (3.7)	−2.2 (3.6)

All data mean (SD) unless stated.

*pCO*_*2*_ partial arterial pressure of carbon dioxide.

**Fig. 1:**
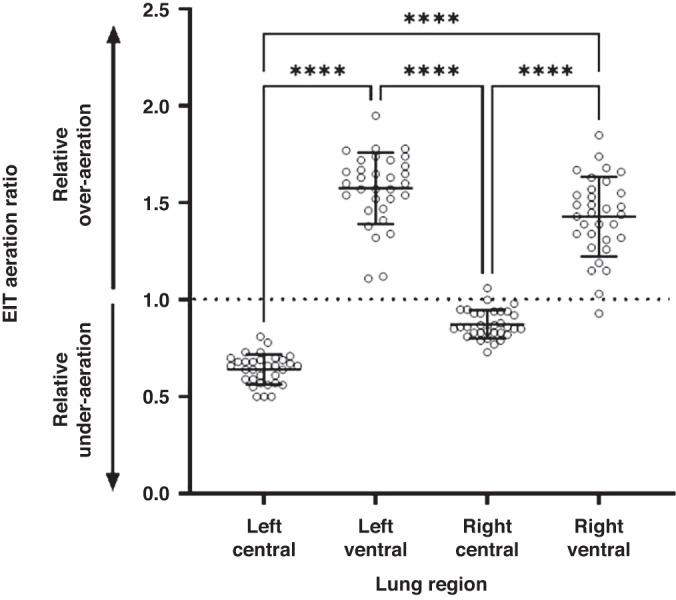
Aeration ratios calculated by EIT across four lung regions of left central, left ventral, right central and right ventral regions. Aeration states represented as a continuous measure and categorized by the following ratio thresholds: <1.0; underinflated with relative under-aeration, 1.0; homogenous aeration, >1.0; relative over-aeration. All data and error bars are represented as mean and standard deviation. Individual circles represent individual EIT measurements. EIT electrical impedance tomography.

Based on EIT, ventral (gravity non-dependent) lung regions were relatively over-aerated whilst dorsal (dependent) regions were atelectatic. Mean (SD) aeration ratios of left central and ventral were 0.64 (0.08) and 1.6 (0.18), respectively, whilst aeration ratios of right central and ventral were 0.87 (0.07) and 1.43 (0.21), respectively (Fig. 1). Mean (95% CI) differences in aeration ratios between the left and right ventral and central regions were 0.93 (0.84–1.03) and 0.56 (0.46–0.65) respectively. Mean differences between left ventral and right central regions and right ventral and left central were 0.23 (0.14–0.33) and 0.79 (0.69–0.88), respectively. Mean differences were also detected between left central and right central and left ventral and right ventral of 0.23 (0.14–0.33) and 0.15 (0.05–0.24), respectively.

**Fig. 2:**
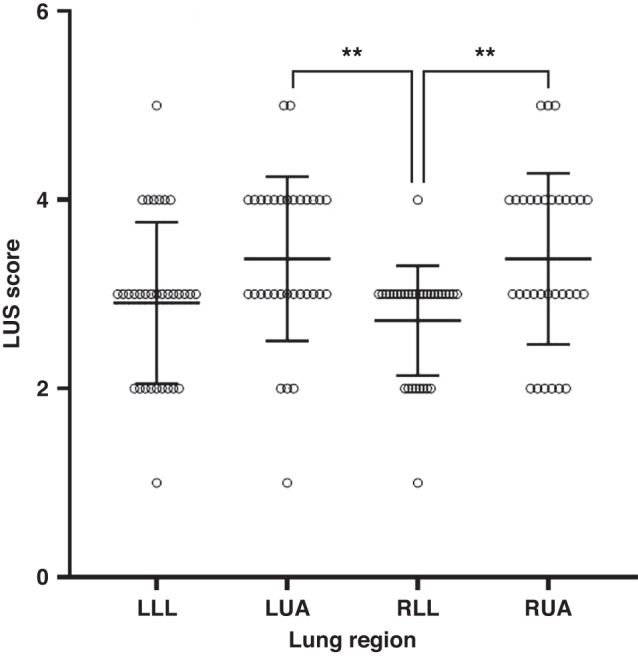
Distribution of lung ultrasound (LUS) scores in four lung regions of left lower anterior (LLL), left upper anterior (LUA), right lower lateral (RLL), right upper anterior (RUA). LUS scores range from 1 to 5. All data and error bars are represented as mean and standard deviation. Individual circles represent individual LUS scores.

Figure 2 shows the individual and distribution of LUS scores of the left and right upper anterior (LUA, RUA) and lower lateral regions (LLL, RLL) bilaterally. Mean (SD) LUS score of LLL vs LUA were 2.9 (0.86) vs 3.4 (0.87), respectively and RLL vs RUA were 2.7 (0.58) vs 3.4 (0.91), respectively. Mean differences (95% CI) in LUS scores between RLL vs. RUA and LLL vs. RLL regions were 0.49 (0.002–0.98) and 0.81 (0.32–1.3), respectively. Mean differences of LUA vs. RLL and RUA vs. LLL regions were 0.62 (0.13–1.1) and RUA vs. LLL of 0.68 (0.19–1.17), respectively.

**Fig. 3:**
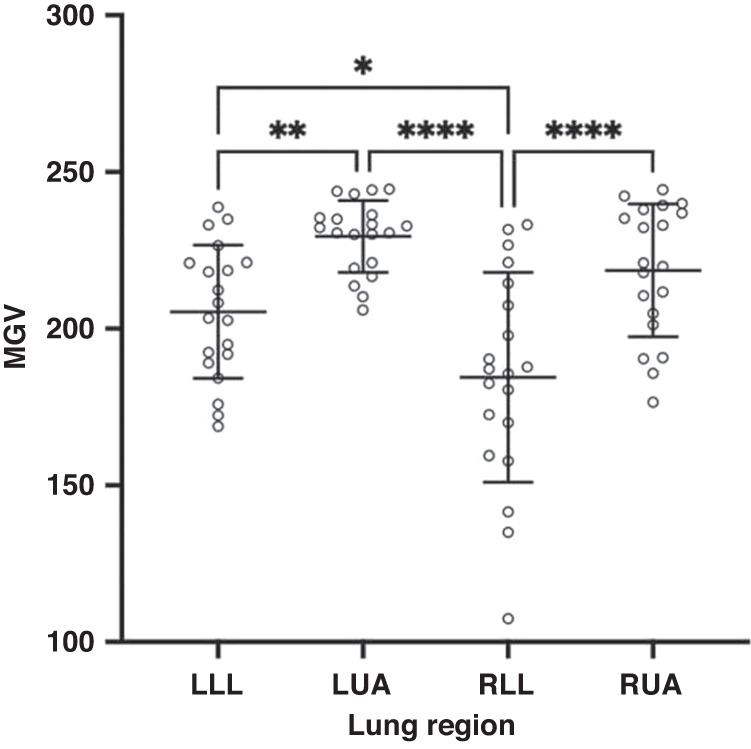
Distribution of MGV calculated by computational image analysis of system 1 across four lung regions of left lower anterior (LLL), left upper anterior (LUA), right lower lateral (RLL), right upper anterior (RUA) regions. *p < 0.05, **p < 0.01, ****p < 0.0001. All data and error bars are represented as mean and standard deviation. Individual circles represent individual MGVs from each individual LUS image. MGV, mean grey value.

Q-LUS_MGV_ detected similar differences in aeration when the analysis was performed on images acquired by System 1 (Fig. 3). Mean (SD) mean grey value (MGV) of LLL vs LUA were 205.4 (21.3) vs 229.4 (11.5) respectively and RLL vs RUA were 184.5 (33.5) vs 218.6 (21.2) respectively. Mean differences (95% CI) of LUA vs. LLL and RUA vs. RLL was 24.3 (4.8–43.3) and 34.1 (14.8–53.4). Mean differences between LUA vs. RLL was 44.9 (25.7–64.2) and LLL vs. RLL was 20.9 (1.63–40.2). All differences were statistically significant.

**Fig. 4:**
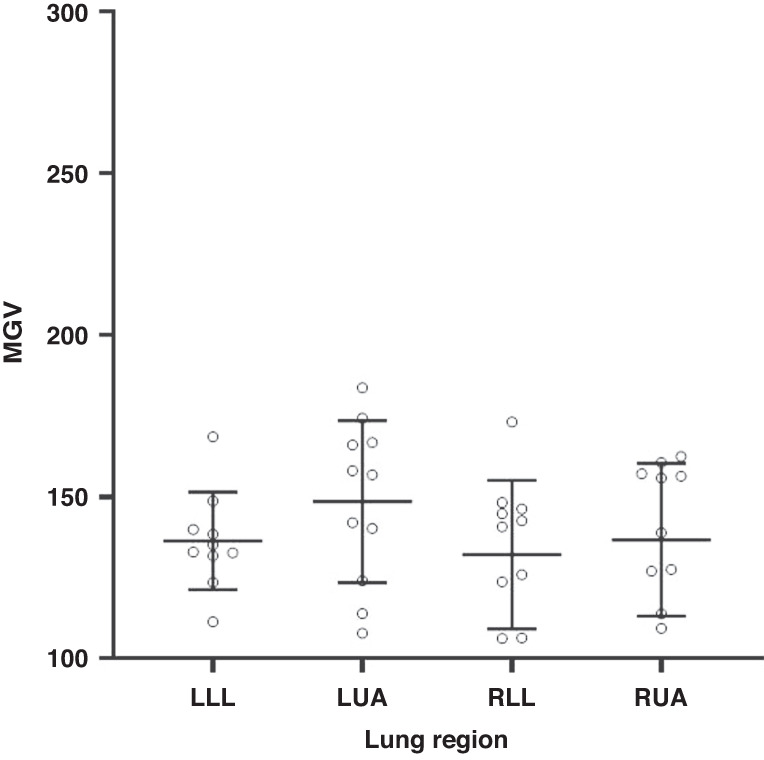
Distribution of MGV calculated by computational image analysis of system 2 across four lung regions of left lower anterior (LLL), left upper anterior (LUA), right lower lateral (RLL), right upper anterior (RUA) regions. All data and error bars are represented as mean and standard deviation. Individual circles represent individual MGVs from each individual LUS image. MGV mean grey value.

Figure 4 represents the MGV derived from images acquired with System 2. Mean (S.D.) MGV of LLL vs LUA were 136.3 (15.1) vs 148.5 (25.0), respectively, and RLL vs RUA were 132.1 (23.0) vs 136.7 (23.6) respectively. Mean differences (95% CI) of LUA vs. LLL and RUA vs. RLL was −12.17 (−38.15–13.81) and −4.60 (−30.0–20.75). Mean differences (95% CI) between LUA vs. RLL was 16.38 (−8.97–41.7) and LLL vs. RLL was 4.21 (−21.8–30.2). Although differences were detected, these were not statistically significant. Overall, values from system 2 were lower compared to system 1.

## Discussion

Mechanically ventilated preterm infants frequently develop inhomogeneous lung aeration^4^ which may contribute to lung injury.^1–3^ EIT reliably detects regional aeration inhomogeneity but is currently predominantly used in research.^5^ There are many advantages of LUS use in preterm infants. They have thinner and smaller thoracic walls, as well as lower lung volumes, which allows for comparatively easier imaging than in adults.^24^ Not only is it an affordable, low-cost technique, but it can also be easily performed at the bedside. Furthermore, despite being operator-dependent, it has high interobserver agreement^25^ with no exposure to radiation, and is able to evaluate changes in the lung via artefacts and patterns over a period of time.^3^ However, its ability to detect regional aeration inhomogeneity requires validation. In our study, we demonstrated that both an expanded visual scoring system^15^ and computer assisted greyscale analysis detected differences in regional aeration in mechanically ventilated preterm lambs.

While visual LUS scores have been extensively researched,^13,14,26,27^ no studies have validated if regional differences in LUS scores reflect inhomogeneous aeration. Regional inhomogeneity, indicated by differences in aeration distribution between gravity dependent and non-dependant peripheral lung regions, is common^4^ and may be associated with lung injury.^28^ In our study, LUS scores were lower in the gravity dependent regions, corresponding with reduced aeration states detected by EIT. This finding may be explained by the influence of gravity,^3^ fluid clearance^29^ and duration of mechanical ventilation.^19^ Furthermore, higher LUS scores were detected in the non-dependent lung regions. The gravity non-dependent regions are known to be easier to ventilate and aerate in states of acute lung disease.^19,20,30^ Overall, the differences in non-dependent and dependent regions represent marked inhomogeneity in our lambs. Our findings suggest that LUS can be used to further characterise aeration distribution beyond its already established use in diagnosing lung pathologies such as pneumonia, pneumothorax, pleural effusion^12^ and its diagnostic use in RDS.^11,24,31,32^

The distribution of LUS scores was wide, suggesting reduced ability to detect small changes in lung volume.^15,24^ Despite this, the artefacts seen are well established in recognising the spectrum of normally aerated to atelectatic lung,^9,12,33^ which correlates to a high versus lower LUS score respectively. Techniques which objectively measure image characteristics may better discriminate smaller changes that are not captured by current scoring systems.^15^ Therefore, we also assessed LUS images using computer assisted image analysis of the mean greyscale value of the pleural region (Q-LUS_MGV_). Computer assisted image analysis of ultrasound images is not new and has been reported previously in preterm infants. In a recent study, Raimondi et al.^34^ recognised that there was a significant correlation with grey scale analysis of LUS images with oxygenation status of infants with respiratory distress. Similar techniques have been used to assess foetal lung maturity and sonographic brain abnormalities in preterm infants.^35,36^ Furthermore, we have recently demonstrated the Q-LUS_MGV_ can detect relatively small changes in lung volume in preterm lambs.^17^ In this study, Q-LUS_MGV_ derived from images of system 1 detected regional aeration differences, represented by higher and lower Q-LUS_MGV_ measurements in the non-dependent and dependent lung respectively. Furthermore, the magnitude of relative differences detected by Q-LUS_MGV_ were larger than that detected by LUS scores. Interestingly, although differences in Q-LUS_MGV_ derived from system 2 were observed, these were not statistically significant. This may be due to the lower number of images acquired using system 2, resulting in inadequate statistical power. More importantly, this finding suggests that direct measures utilising greyscale analysis may not be interchangeable between ultrasound systems. Our findings suggest that Q-LUS_MGV_ may have potential to improve the sonographic detection of aeration inhomogeneity, but further work is needed to refine this technique so that it is interchangeable between ultrasound systems.

CT is the gold standard reference method to measure lung aeration,^16,37^ however, this was not possible as the preterm lambs in this study were on placental support. Furthermore, CT imaging is not routinely performed in preterm infants. However, LUS scoring has demonstrated strong correlations when compared to CT as an imaging tool in predicting severity of adult lung pathologies,^38^ and LUS is validated in facilitating diagnosis and prognosis of adult RDS patients.^39^ As EIT measures of lung aeration have been validated against CT, EIT was chosen for comparative imaging.^40^ LUS regions were categorised as upper anterior and lower lateral, which correlate with EIT’s cross sectional ventral and central regions, respectively.^6^ In our study, differences in LUS scores corresponded with differences in aeration measured by EIT, confirming the ability of LUS to detect changes in regional aeration.

The ability to accurately detect inhomogeneous lung aeration at the bedside may help better understand the development of chronic lung conditions such as BPD. Recent studies have demonstrated that LUS and EIT may accurately predict a diagnosis of BPD.^22,27,29,41^ However, LUS may not provide additional accuracy over traditional predictors such as gestational age and duration of mechanical ventilation.^41^ No measures of lung injury were performed in this study; thus, we limited our objectives to aeration states and not resultant injury. Despite this, we contend that to better understand disease development, tools which can continuously monitor aeration changes, particularly regional changes for inhomogeneity, are required. This new application of LUS may help detect and monitor aeration inhomogeneity and resultant lung injury in preterm infants, warranting validation studies in human infants.

Our study has limitations. Preterm lambs in this study were on placental support for longer than standard delayed cord clamping. This could potentially impede lung liquid clearance and influence aeration. Furthermore, results from animal studies have limited generalisability to humans, especially when breathing is suppressed. However, the ventilated preterm lamb is a well-established analogue of the preterm human lung.^21^ This was a single observer study, however, we have previously demonstrated that our scoring system has excellent interobserver agreement^15^ and clinical scoring systems have good sensitivity and specificity for a number of respiratory disorders.^9,11,13^ Despite differences in LUS scores being statistically different, they were widely distributed. A previous study by ref. ^16^ has demonstrated that although global and regional LUS were strongly associated with lung tissue density, LUS is unable to detect small changes in lung volume. However, our large sample size provided adequate power to detect relatively small changes in LUS scores, whereby the scoring system has been validated against absolute in-vivo measures of lung volume in preterm lambs.^15^ Finally, image analysis of the LUS images was not done in real time, precluding use in current clinical practice. Additionally, Q-LUS_MGV_ measures were not consistent between ultrasound systems, demonstrating an important limitation of this evolving technique. Future efforts to refine and automate these measurements are required to facilitate practical use at the bedside. EIT is not without limitations as a measure of relative aeration. These have been reported in detail previously.^6,19^

## Conclusion

LUS reliably detects regional aeration inhomogeneity compared to EIT. Our findings suggest that LUS may be able to detect regional differences in lung aeration in preterm infants and may be used to better understand the evolution of lung pathology. Validation of these findings in human infants is warranted.

### Supplementary information

Supplementary File

## Data Availability

Individual animal data collected during the study and statistical analysis will be available beginning 3 months and ending 23 years after article publication to researchers who provide a methodologically sound proposal with approval by an independent review committee. Data will be available for analysis to achieve aims in the approved proposal. Proposals should be directed to arun.sett@mcri.edu.au; to gain access, data requestors will need to sign a data access or material transfer agreement approved by the Murdoch Children’s Research Institute.
