# Supplementary material for: Lung ultrasound detects regional aeration inhomogeneity in ventilated preterm lambs
Source: Pediatr Res. 2023 Aug 17;95(1):129–34. doi: 10.1038/s41390-023-02781-1 (PMC10798896; doi:10.1038/s41390-023-02781-1)
Supplement: Supplementary file 1 — Supplementary File [file 41390_2023_2781_MOESM1_ESM.pdf]

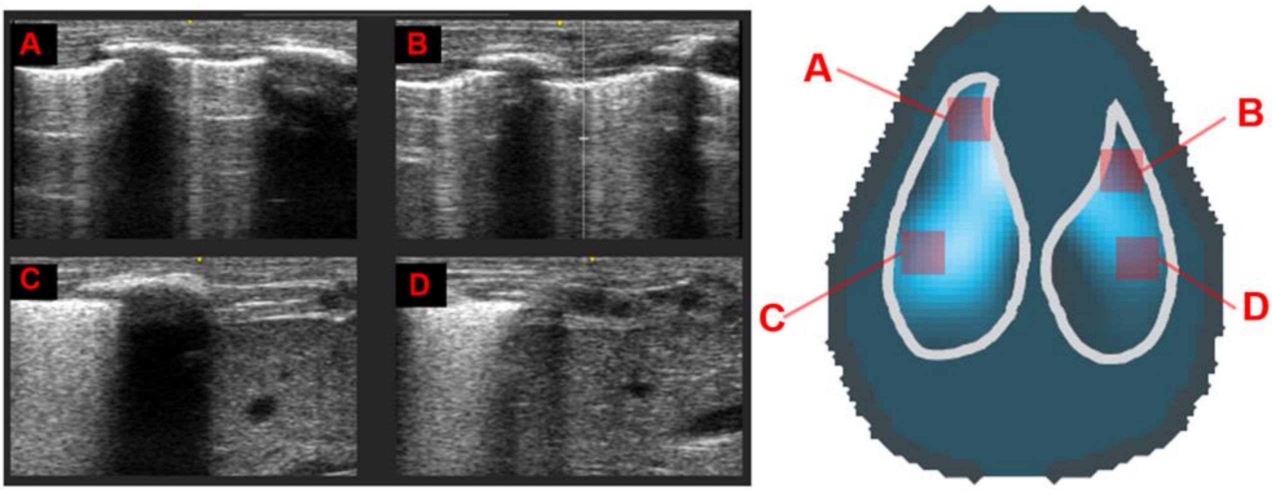

Supplementary Figure 1 legend:

Supplementary Figure 1. Heterogeneous lung aeration represented by lung ultrasound (LUS) on the left and a functional electrical impedance tomography (fEIT) image from a lamb lung on the right, labelled (A) Right Upper Anterior region (on LUS) corresponding to Right Ventral region (on EIT). (B) Left Upper Anterior (LUS) vs Left Ventral (EIT). (C) Right Lower Lateral vs Right Central (EIT). (D) Left Lower Lateral vs Left Central (fEIT). fEIT relative gas volume within the lung regions represented using a heat map with dark blue indicating the least gas volume and white/lightest blue the most gas volume for the imaging period.
